# Mechanisms of microbiota modulation: Implications for health, disease, and therapeutic interventions

**DOI:** 10.1097/MD.0000000000038088

**Published:** 2024-05-10

**Authors:** Okechukwu Paul-Chima Ugwu, Esther Ugo Alum, Michael Ben Okon, Emmanuel I. Obeagu

**Affiliations:** a Department of Publication and Extension, Kampala International University, Kampala, Uganda.

**Keywords:** gastrointestinal disorders, gut microbiota, immune-mediated diseases, microbial communities, Microbiota modulation, safety considerations, therapeutic interventions, translational research

## Abstract

Microbiota modulation, the intentional change in the structure and function of the microbial community, is an emerging trajectory that holds the promise to mitigate an infinite number of health issues. The present review illustrates the underlying principles of microbiota modulation and the various applications of this fundamental process to human health, healthcare management, and pharmacologic interventions. Different strategies, directing on dietary interventions, fecal microbiota transplantation, treatment with antibiotics, bacteriophages, microbiome engineering, and modulation of the immune system, are described in detail. This therapeutic implication is reflected in clinical applications to gastrointestinal disorders and immune-mediated diseases for microbiota-modulating agents. In addition to this, the review outlines the challenges of translating researched outcomes into clinical practice to consider safety and provides insights into future research directions of this rapidly developing area.

## 1. Introduction

**Figure 1.**
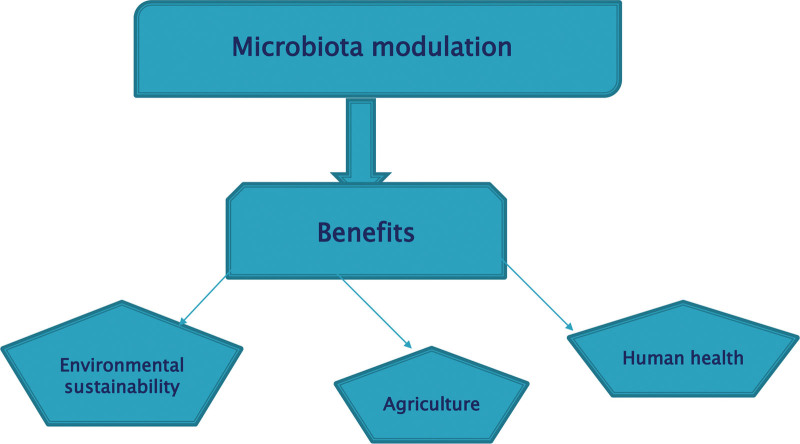
Benefits of microbiota modulation

Microbiota modulation, the newly emerging frontier, based on conscious changes in microbial community composition and function, offers a wide range of benefits in human health, agriculture, and environmental sustainability as shown in Figure 1.^[1,2]^ This critically important field involves a myriad of approaches from dietary modifications to the sophisticated microbiome engineering techniques.^[3]^ In the field of gastrointestinal diseases great achievements have been made in diagnostic accuracy and therapy development, which allowed for personalized medicine. Besides, microbial-modulating agents can provide a new immune mechanism, providing remedies for imbalanced immune system and autoimmune disorders.^[4]^ Consequently, research translation into clinical practice encounters a myriad of difficulties, which therefore demands a unified strategy to accommodate protocol standardization, treatment optimization and prolonged safety surveillance.^[5]^ In addition, microbiota manipulation impact is not just limited to the gut health as it also acts beyond this by influencing neurological disorders, metabolic syndrome, infectious diseases, and regulatory landscapes.^[6]^ Along with walking through the complicated issues, living up to the collaboration among the stakeholders, including researchers, healthcare providers, industry partners, and regulatory agencies to unclog the potential of modulating human microbiota to enhance health and general wellbeing is fundamental.^[7]^ The complex interplay of the gut microbiota and neurodevelopmental processes highlights the quintessential role of the gut microbiota in neurological disorders that include depression, anxiety, Parkinson disease, and autism spectrum disorders.^[8]^ New research seems to indicate that influencing on the gut microbiome through approaches like dietary improvement, probiotics, and fecal microbiota transplantation could be a key factor in dealing with the mood swings, behavior issues, and cognitive decline seen in these conditions. The gut-brain axis represents a promising tool for developing neurotherapeutic solutions that aim for the microbiota as gateways to the management of neurological disorders and improvement of the quality of life for the affected individuals.^[9]^ The disruption of a proper balance in the gut microbiota is intimately connected to the development of the metabolic syndrome, insulin resistance, and obesity, which are essentially the main causes of cardiovascular disease and type 2 diabetes.^[10]^ Interventions that modulates the gut microbiota through dietary modifications, microbial therapies, and other interventions directed to specific targets have a promise to fight multifaceted conditions and their associated comorbidities.^[11]^ Through microbial rehabilitation and metabolic homeostasis restoration, microbiota modulation approaches provide a very optimistic direction for overcoming the worldwide metabolic syndrome and its related health problems.^[12]^ Microbiota manipulation, particularly through strategies that involve for instance probiotics, prebiotics and fecal microbiota transplantation is a relatively new path in preventing and treating infectious diseases, caused by different pathogens such as viruses, bacteria, and fungi.^[13]^ These interventions amplify the host immune system, solidify the mucosal barrier, and disrupt pathogen colonization, hence, imparting resistance to infectious organisms.^[14]^ Employing the advantages of microbiota modification would increase the efficiency of the current infectious disease management methods and challenge the new infectious risks at a global level.^[15]^ Although microbiota-manipulating compounds bring the possibility of treatment, safety concerns and adverse reactions (especially among those that are vulnerable) should be considered first of all.^[16]^ Comprehensive monitoring and control are absolutely vital to assure the safety and effectiveness of these tools in the clinical environment. Comprehensive risk-benefit assessments, robust post-market surveillance, and adherence to the stringent quality standards could help eliminate the risks and to extract the immense therapeutic benefits from microbiota modulation across different patient populations.^[17]^ The constantly changing scenario of microbiota manipulation brings in an era of great inventions, which are used for the treatment of a lot of diseases and health conditions. In the future, microbiome-based therapies, phage therapy, microbiome engineering, and the making of microbiota-targeted drugs will be among the directions for research.^[18]^ Through these innovative solutions, high specificity to disease pathogenesis and different health conditions can be achieved by microbiota alteration that leads to optimized treatment outcomes via personalized interventions.^[19]^ Regulatory barriers and market dynamics matter significantly for the prospective microbiota-altering drugs to get on the market.^[20]^ Undertaking safety, efficacy, and quality while underwriting reimbursement policies becomes difficult and requires partnership work of all the industrial players, regulatory authorities, health care providers, and researchers. The creation of regulatory landscape assimilatable to the development of microbiota modulation is necessary in order to promote innovation, provide patients access to novel therapy, and prop up the sustainable development of this thriving field.^[21]^ Microbiota manipulation takes a leading position because of its revolutionary nature in healthcare innovations allowing for disease management please people all over the world.^[22]^ The impact of microbiota modulation on, for example gastrointestinal disorders, neurological conditions, metabolic syndrome, and infectious diseases extends to many domains of health and medicine. Nevertheless, maximizing the opportunities of microbiota regulation hinges upon various measures to overcome issues linked to standardization, optimization, safety, and oversight.^[23]^ The science of microbiota modulation can transform healthcare by making use of its expeditious research, integrating collaboration, and embracing novel strategies.^[10]^

## 2. Methodology

### 2.1. Scope

This narrative review aims to provide a comprehensive overview of the mechanisms underlying microbiota modulation and its implications for human health and therapeutic interventions. The scope encompasses various approaches to microbiota modulation, including dietary interventions, fecal microbiota transplantation, antibiotic therapy, bacteriophages, microbiome engineering, immune system modulation, and their clinical applications in gastrointestinal disorders and immune-mediated diseases.

### 2.2. Literature search strategy

A comprehensive literature search was conducted using electronic databases such as PubMed, Scopus, Web of Science, and Google Scholar. Keywords including “microbiota modulation,” “gut microbiota,” “therapeutic interventions,” “immune-mediated diseases,” and “gastrointestinal disorders” were used in various combinations to identify relevant articles published in peer-reviewed journals. Additionally, reference lists of selected articles were manually searched to identify additional relevant studies.

### 2.3. Inclusion criteria

Articles included in this narrative review were original research articles, reviews, and meta-analyses published in peer-reviewed journals. Articles focusing on mechanisms of microbiota modulation and their implications for human health, as well as those discussing therapeutic interventions targeting microbiota modulation in gastrointestinal disorders and immune-mediated diseases, were included.

### 2.4. Exclusion criteria

Articles were excluded if they did not meet the inclusion criteria, were not written in English, or were duplicate publications. Additionally, articles focusing solely on animal studies or in vitro experiments without relevance to human health were excluded.

### 2.5. Categorization of literature

Selected articles were categorized based on the type of microbiota modulation strategy discussed (e.g., dietary interventions, fecal microbiota transplantation, antibiotic therapy) and their clinical applications in gastrointestinal disorders and immune-mediated diseases.

### 2.6. Critical analysis

A critical analysis of the synthesized findings was conducted to evaluate the strengths and limitations of the existing literature, identify gaps in knowledge, and discuss the implications for clinical practice and future research.

### 2.7. Interpretation and discussion

The synthesized findings were interpreted in the context of existing literature and discussed in terms of their implications for human health, disease management, and therapeutic interventions targeting microbiota modulation.

### 2.8. Reporting

The findings of this narrative review are reported clearly and concisely, with appropriate referencing of sources to ensure transparency and accuracy.

### 2.9. Ethical considerations

This narrative review adheres to ethical principles governing research conduct, including proper citation of sources and adherence to journal guidelines and publication ethics. Additionally, ethical considerations related to the use of human participants in original research studies included in this review were carefully considered and addressed.

### 2.10. Mechanisms of microbiota modulation

**Figure 2.**
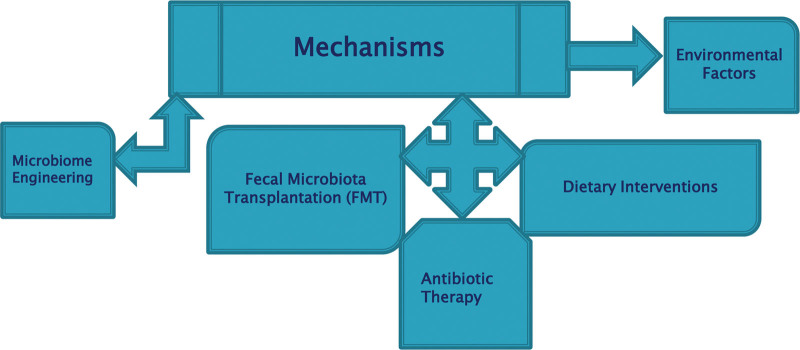
Mechanisms of microbiota modulation

Deliberate alteration of microbial community structure and/or function in a particular environment such as the human gut, skin, or soil refers to modulation of the microbiota. This modulation can have important repercussions for health, agriculture, and environmental sustainability.^[1]^ Microbiota has several mechanisms through which it can be modulated; natural, induced, or engineered as shown in Figure 2. There are several approaches to achieving microbiota modulation including:

### 2.11. Dietary interventions

• Prebiotics: These are not digestible dietary components with examples like fiber inulin and oligosaccharides that stimulate selectively growth and/or activity of good bacteria inside the gut. They act as specific microbes’ substrates thereby enhancing their proliferation and metabolic activity.^[2]^
• Probiotics: Live microorganisms that on consumption at adequate levels confer a benefit on health. Probiotic sources include fermented foods such as yogurt or kimchi or they can be taken as supplements. They introduce beneficial species directly into the gut microbiota or affect the activity patterns of existing populations within it.

Synbiotics are combinations of prebiotics and probiotics which have synergistic effects on the gut microbiota. In the gut, prebiotics provide food for probiotics thus hiking up their presence and activity.^[3]^

### 2.12. Fecal microbiota transplantation (FMT)

FMT is where fecal material is taken from a healthy donor and given to someone whose microbiota has been disrupted or become unhealthy as a result of disease. Despite being mainly used for recurrent Clostridioides difficile infections, FMT also looks into other conditions linked with dysbiosis like metabolic disorders, and inflammatory bowel disease (IBD). FMT brings back the diversity of microorganisms in an individual’s intestine hence promoting better health outcomes.^[4]^

### 2.13. Antibiotic therapy

Although antibiotics are meant mainly to kill pathogenic bacteria, they can have considerable impacts on commensal microbiota. Antibiotics may result in dysbiosis when susceptible species are killed or it may change the balance between different microbial taxa. Dysbiosis following such disruptions increases vulnerability to opportunistic infections and other diseases related to microbiome disturbance. Yet at times, antibiotics can be exploited intentionally to influence the composition of microbiota, such as small intestinal bacterial overgrowth or specific depletion of pathogens.^[3]^

Bacteriophages are also known as bacterial viruses which invade and multiply in bacteria. They serve as natural predators of bacteria that form microbial communities and play an important role in determining the population. The phage therapy aimed at curing the bad things in microbial, one of the possible directions regarding the modulation of microbiota is being considered. Phage therapy shows promise as an effective treatment, especially with the capacity to eradicate harmful bacteria while still preserving beneficial indigenous microbiota present in different environments such as the human body.^[4]^

### 2.14. Microbiome engineering

Under this approach, microbial communities are deliberately influenced or triggered through the solutions and techniques of synthetic biology. It includes methodologies like designing probiotic bacteria to transmit curative molecules or manipulating microbial consortia with distinctive roles. The great potential of engineering the microbiome allows us to develop precise and controlled interventions that modulate microbial nutrients or species for a range of uses, such as disease control, and environmental remediation, among other bioproduction applications.^[3]^

### 2.15. Environmental factors

Several environmental variables have been put forward to influence the composition and function of microbial communities, including stress, pollution, dietary changes, and lifestyle. Since the factors that shape microbiota are very crucial to understanding how these processes lead to changes in the microbiome, it is important to understand them to solve them using appropriate measures. For instance, direct effects may be produced by environmental pollutants or dietary components on the growth and metabolism of microorganisms leading to changes in compositional traits and activity of microbiota.^[4]^

1. Immune System Modulation: The host immune system helps in the development of an active microbiota and also guides the composition and functions of different structures. Indirectly, the immunomodulatory therapeutics impact on microbiota and their hosts; with immunosuppressants or immunostimulant types. For instance, modulation of the gut immune system through dietary components or pharmacological agents influences the composition of gut microbiota while one way or another it is also influenced by way of composition of the same.^[3]^
2. Microbial Interactions: Within a community, microorganisms can interact with each other in different ways such as through competition whereby some organisms feed on others thus supporting the reproduction of these species. Thus comprehension of these microbial relationships is mandatory for providing an opportunity to predict how e given intervention affecting the modulation of the microbiota affects structure and functionality in general. For instance, a newly introduced phototrophic microbial species in the eco-system of the microbiota through probiotics or phage therapy might lead to ecological changes that affect other community members.^[4]^
3. Host-Microbe Crosstalk: The messages circulating along various signaling pathways enable two-way communication between the host and its associated microbiota. These interactions, facilitated by host-derived molecules as well as contents and microbial metabolites discussed are key to the host homeostasis and how it affects the microbe’s Kingdom. There are many ways to consider host-microbe crosstalk either through dietary interventions, microbial therapies, or host-targeted therapies; modifications in the composition and functions of microbiota shall also be possible.^[3]^
4. Ecological Principles: Secondly, microbial communities operate on advanced ecological rules that include niche separation, succession, and resilience. Considering these ecological principles is essential to devising balanced approaches toward modulating the microbiota. For instance, in the case of rebuilt microbial diversity or stabilized ecosystems, the interventions improve perturbation tolerance and support for long-term equilibrium between the microbiota. Microbiota modulation mechanisms are dynamic and complex, as they involve various dietary interventions, microbial therapies, ecological principles, and host-pathogen activity. Microbiota modulation is effective when this body of knowledge is fully developed and targets microbiota across localized environmental conditions has the body in human beings, agricultural systems, or natural surroundings.^[4]^

### 2.16. Clinical applications in gastrointestinal disorders: a comprehensive review

The gastrointestinal tract is arguably the guts of the human body; its proper function encompasses digestion, absorption, and elimination of nutrients hence a functional GI system translates to whole organismic health. Such disorders as gastroenterological pathologies have numerous pathogenic conditions influencing the architecture and or function of the digestive passage that cause an extensive diversity of symptoms, from slight discomfort to debilitating ailments. These diseases are responsible for major clinical problems due to their complex etiology, or a wide range of symptoms and activities when they respond to treatment among people.^[5]^ Detecting the past pathophysiology, diagnosis, and management of gastrointestinal disorders gave approximately 7 relevant findings in recent years. Novel diagnostic measures such as those associated with imaging techniques and molecular biomarkers have allowed a higher sensitivity for the detection of conditions on the one hand, but also follow-up regarding conditions, among others. In addition, forms such as microbiota-based treatments and targeted biologics have become a practical approach regarding the setting of successful results for resistant disorder.^[6]^ Inflammatory Bowel Diseases (IBD): Classic male members of the IBD group include Crohn’s disease and ulcerative colitis – a chronic inflammatory disorder type that has reversible episodes of intestinal inflammation. Among these complex disorders which involve semantic corruption, are evils such as severe morbidity that leads to strictures, fistulas, and colorectal cancers. The treatment of IBD is a multi-disciplinary form of treatment with various intervention modalities including surgery or nutrition.^[7]^ In the treatment of IBD, recent attempts have mainly shifted toward prescribing types of early and intensive therapies that form induction to not only amass completion but also reduce corticosteroids. The use of immunosuppressive agents targeting specific cytokines like TNF-αwith biological therapeutics such as anti-TNF antibodies is a revolutionary change in the landscape of therapy regarding IBDs because these drugs provide better results and far fewer toxic side effects compared to older medications.^[8]^

Moreover, treatment strategies for PMSA-related cancers that are primarily based on the genetic and serological biomarkers presented prospective possibilities in facilitating decision-making concerning treatment and forecasting disease outcomes. In the sphere of surgical management, minimally invasive techniques including the laparoscopic approach and robotic-assisted surgery are recommended more in managing IBD-related adverse events as they involve lower levels of postoperative pain after the maintenance period is over along with decreased length of hospital stay and accelerated recovery time. In addition, intestinal tissue engineering and stem cell therapies have a great prospect to give rise to the tissues’ regeneration as well as customized surgical remedies for patients with intractable monad. Gastroesophageal Reflux Disease (GERD): Gastroesophageal reflux disease is a widespread gastrointestinal illness that involves the constant development of gastric content from traveling backward, into the esophagus presenting symptoms such as heartburn, regurgitation, and dysphagia. GERD comprises a wide range of clinical features from occasional mild symptoms to severe erosive esophagitis and Barret’s esophagus intermediate step into developing advanced headless adenocarcinoma. GERD management entails the progressive approach beginning with non-pharmacological therapy in the form of dietary modifications and lifestyle adjustments, then pharmacotherapy using PPIs or H2RAs for symptom control.^[8]^ On the other hand, a group of patients may develop highly resistant symptomatology medical treatment and therefore these cases require additional investigation via endoscopic and/or surgical procedures. Various endoscopic modalities like radiofrequency ablation (RFA) and endoscopic mucosa resection (EMR) came into existence because they provide effective therapy in cases of Barrett’s esophagus and early neoplasia esophageal with maintained eradication of the dysplastic tissue without affecting the normal functioning of the esophagus.^[7]^ Moreover, many minimally invasive surgical procedures including laparoscopic fundoplication have shown effectiveness in patients with GERD who failed medical treatment or have an allergy to long-term PPI use.

#### 2.16.1. Irritable Bowel Syndrome (IBS)

Its main symptoms are abdominal pain and hardening, a change in bowel habits, and bloating without structural changes. The pathophysiology of IBS is complex, which encompasses dysregulation of the brain-gut axis and visceral hypersensitivity that involve muscarinic receptors within the intestinal wall as well as altered gut motility dynamics in combination with the increased gastrointestinal permeability caused by micro-organisms alterations (dysbiosis). The management of IBS is a symptomatic control that restores quality of life with dietary adaptations, pharmacotherapeutic products, and psychological interventions. Various dietary interventions, such as a low FODMAP (fermentable oligosaccharides, disaccharides, monosaccharides, and polyols) diet appear effective in alleviating symptoms in some patients by minimizing fermentation precursors associated with indigestion complaints. Interfering pharmacological treatment of IBS encompasses the domains of certain symptoms achieved using, antispasmodics, antidepressants, and probiotics that have various response rates between the subjects. Besides, various psychological interventions including CBT and gut-targeted hypnotization therapy have been shown to work when dealing with psychosocial issues IBS and symptom management.^[7]^

#### 2.16.2. Gastrointestinal malignancies

Gastrointestinal cancers, collectively encompass the whole group of cancerous tumors that have originated from multiple anatomical sites within the gastrointestinal tract such as the esophagus, stomach, liver, pancreas colon, and rectum. And, these types of cancers constitute one of the key challenges in the world health system and have high rates of morbidity or mortality.

Since advanced technologies for gastrointestinal malignancy management have been created, there were advances in areas dealing with survival outcomes by multimodal approaches that involved surgery chemotherapy radiation therapy, and targeted therapies. Earlier detectable colorectal and esophageal cancers have undeniably been made accessible through the use of screening programs relying on endoscopic techniques such as colonoscopy and EGD, thereby facilitating interventional treatment at early stages of diseases. Today, the concept of precision medicine is based on molecular profiling and targeted therapies that transformed treatment paradigms for gastrointestinal malignancies including tumors manifesting characteristics such as HER2 amplification in gastric cancer or RAS mutation in colorectal neoplasms. Biological therapies have also led to innovative immunotherapeutic treatment agents in some cases, including checkpoint inhibitors that disrupt immune response pathways and target the T lymphocytes that mediate tumor-induced immunity; these show effectiveness in subsets of gastrointestinal cancers as proofs of concept for precision treatments.

#### 2.16.3. Functional gastrointestinal disorders

The term, functional gastrointestinal disorders is used to describe a family of diverse conditions featuring persistent or intermittent intestinal complaints despite a lack of anatomic distortion and metabolic disarrangements. The disorders of interest in this paper include functional dyspepsia, functional constipation, and the syndrome named functional abdominal pain syndrome which together are highly prevalent having a huge potential for lowering quality of life.^[7]^

### 2.17. The immunomodulatory effects of microbiota-modulating agents and their role in immune-mediated diseases

The human microbiota, encompassing bacteria, viruses, fungi, and other microorganisms, constitutes a dynamic ecosystem that significantly influences human health and disease. Among its diverse functions, the microbiota plays a crucial role in educating and regulating the immune system. Perturbations in microbiota composition, termed dysbiosis, have been linked to various immune-mediated diseases, including autoimmune disorders characterized by aberrant immune responses against self-antigens, and allergies marked by hypersensitivity reactions to harmless environmental substances.^[8]^

### 2.18. Microbiota and immune homeostasis

The gut microbiota, in particular, exerts profound effects on immune development and function. Commensal bacteria within the gut lumen interact with the host’s immune cells, such as dendritic cells, macrophages, and T cells, influencing their activation, differentiation, and effector functions. Key mechanisms include the production of short-chain fatty acids (SCFAs), modulation of regulatory T cells (Tregs), and maintenance of intestinal barrier integrity. Disruption of this delicate balance can lead to immune dysregulation and the onset of immune-mediated diseases^[8]^

### 2.19. Microbiota-modulating agents

Various strategies have been explored to modulate the microbiota composition and restore immune homeostasis in the context of immune-mediated diseases. These include dietary interventions, probiotics, prebiotics, antibiotics, fecal microbiota transplantation (FMT), and microbial metabolites. Each approach targets specific aspects of microbiota-host interactions, aiming to promote beneficial microbial communities while suppressing pathogenic species.^[9]^

### 2.20. Immunomodulatory mechanisms

Microbiota-modulating agents exert their effects through diverse immunomodulatory mechanisms. Probiotics, for instance, can directly interact with immune cells via surface molecules, secreting anti-inflammatory cytokines and promoting Treg differentiation. Prebiotics selectively stimulate the growth of beneficial bacteria, leading to the production of SCFAs that modulate immune cell function. FMT introduces a diverse microbial community into the recipient’s gut, restoring microbial balance and immune homeostasis.^[10]^

### 2.21. Role in autoimmune disorders

The link between microbiota dysbiosis and autoimmune diseases, such as rheumatoid arthritis, inflammatory bowel disease, and multiple sclerosis, has been extensively investigated. Emerging evidence suggests that microbiota-modulating agents hold promise as adjunctive therapies in managing autoimmune disorders by attenuating inflammatory responses and promoting immune tolerance.^[11]^

### 2.22. Role in allergies

Many studies also suggest the involvement of modifications in microbiota composition in cases of allergic disorders which include asthma, atopic dermatitis and food allergies. Modulation of the microbiota proved a viable strategy to reduce rates of allergic sensitization as well by reducing levels of serum IgE and improving intestinal barrier function in infants after increasing protective probiotics via early supplementation during pregnancy and lactation.

### 2.23. Clinical implications and future directions

In the last 2 decades, substantial work was done about immune-mediated diseases with microbiota-modulating agents but despite having a large number of supporting evidence for immunomodulatory effects built around the same there still exists some challenges. To translate these findings into clinical practice, it would be necessary for standards to be developed regarding intervention protocols to determine the best treatment formulations and to uncover patient-specific factors affecting responsiveness. Microbiota – immune interactions level, along with the personalization possibilities in future therapies as well as long-term monitoring of efficacy and safety should be considered hereafter.^[10]^

Microbiota is essential in regulating immune reactions and its disorder affects the incidence of several immunological diseases ranging from autoimmune diseases to allergies. The pharmacological modulation of microbiota by microbiota-modulating agents is a new but promising therapeutic avenue as it balances the microbial flora and brings system balance at large for better host health. More work needs to be done to understand the mechanisms behind their functioning and how best to harness them for maximum therapeutic effectiveness in the treatment of immune-mediated diseases.

### 2.24. Modulation of microbiota influence on depression, anxiety, Parkinson disease, and autism spectrum disorders

All outcomes of neurodevelopmental processes are also reflected in the development of neurodegenerative mechanisms, and it is more prominent following changes caused by the gut microbiota. The gut microbes’ colonization in early infancy is one of the core determinants for determining CNS maturation and structuring emotional regulatory, social behavior as well as motor circuits.^[11]^ Those effects of modulation through microbiota are also examined in the preclinical and clinical studies on depression and anxiety. Treatments that modify gut microbiota are shown to deal better with neurological signs related to depressive disorder. Plans of microbiota manipulation involve diet modification, probiotics; FMT, and Repletion with microbial metabolites. All in all, it has been found that these interventions can improve the mood and behavior of affected persons and reduce motor symptoms as well as reinforce cognitive functions.^[12]^ Several advances in this field include the microbiota-based therapeutic approaches which hold a great deal of potential for better management of neurological disorders. Nevertheless, numerous difficulties persist regarding the elaboration of individualized interventions that are adjusted to specific microbiota profiles and which can be appropriately optimized when used. Also, there is a necessity for long-term assessments of safety and efficacy parameters. In addition, the detailed mechanisms involved in microbiota-brain interactions are necessary for defining therapies that directly address these relationships by targeting treatment opportunities with high therapeutic potential.^[13]^ The gut-brain connection plays an important part in the pathophysiology of neurological disorders, and this failure is due to alterations within the structures formed by microbiota dwelling under undigested materials from ingestion. However, modulation of the gut microbiota using different modes of intervention opens a new avenue to managing neurological symptoms and also alleviates patient outcomes. More studies are needed to explore the mechanisms and should be conducted targeting microbiota-based therapies for individualized therapy of central nervous system diseases.^[14]^

### 2.25. The potential of microbiota-modulating agents in alleviating metabolic syndrome, insulin resistance, obesity, and associated comorbidities

The role of gut microbiota in human health has garnered significant attention in recent years, and researchers have been exploring the potential of microbiota-modulating agents in addressing various health conditions, including metabolic syndrome, insulin resistance, obesity, and associated comorbidities. The gut microbiota refers to the diverse community of microorganisms residing in the gastrointestinal tract, including bacteria, viruses, fungi, and archaea.^[15]^

### 2.26. Metabolic syndrome

Metabolic syndrome is a cluster of conditions that often occur together, including abdominal obesity, high blood pressure, elevated blood sugar levels, and abnormal lipid profiles. Studies suggest that the composition and activity of the gut microbiota play a role in metabolic syndrome. Dysbiosis, or an imbalance in the gut microbial community, has been associated with inflammation and insulin resistance key components of metabolic syndrome.^[16]^

### 2.27. Insulin resistance

Insulin resistance occurs when the body’s cells become less responsive to the effects of insulin, leading to elevated blood sugar levels. Some research indicates that the gut microbiota can influence insulin sensitivity.^[17]^ Modulating the gut microbiota may impact insulin resistance through various mechanisms, including the production of bioactive compounds and the regulation of inflammation.^[17]^

### 2.28. Obesity

Obesity is a complex condition with multiple factors at play, and the gut microbiota is emerging as a potential contributor. Studies have shown differences in the gut microbial composition between lean and obese individuals. Microbiota-modulating agents may influence energy balance, fat storage, and inflammation, which are all linked to obesity.^[18]^

### 2.29. Comorbidities

Metabolic syndrome, insulin resistance, and obesity are often associated with several comorbidities, including cardiovascular disease, nonalcoholic fatty liver disease, and type 2 diabetes. The gut microbiota’s role in these conditions is an area of active research. Modulating the microbiota may have downstream effects on inflammation, lipid metabolism, and other processes contributing to these comorbidities.^[18]^

### 2.30. Microbiota-modulating agents

Various strategies are being explored to modulate the gut microbiota, including dietary interventions, prebiotics, probiotics, postbiotics, and fecal microbiota transplantation. Prebiotics are substances that promote the growth of beneficial bacteria, while probiotics are live microorganisms with potential health benefits. Postbiotics are bioactive compounds produced by the gut microbiota.^[19]^ While the potential of microbiota-modulating agents is promising, challenges remain. The field is still evolving, and more research is needed to understand the specific mechanisms involved and the optimal strategies for intervention. Factors such as individual variability in microbiota composition and the complexity of interactions between host genetics, diet, and the microbiota add complexity to the research.^[20]^ The gut microbiota appears to play a significant role in metabolic health, and microbiota-modulating agents hold promise as potential interventions for conditions such as metabolic syndrome, insulin resistance, obesity, and associated comorbidities. Continued research is crucial to unravel the intricate connections between the gut microbiota and human health and to develop effective and personalized strategies for intervention.

### 2.31. The role of microbiota modulation in preventing and treating infectious diseases

**Figure 3.**
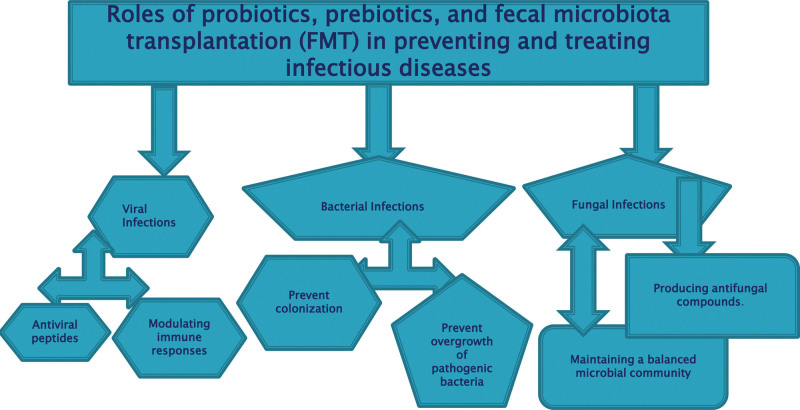
The role of microbiota modulation in preventing and treatment of infectious diseases.

Microbiota modulation, particularly through the use of probiotics, prebiotics, and fecal microbiota transplantation (FMT), has garnered significant attention in recent years for its potential role in preventing and treating infectious diseases.^[21]^ The human microbiota, comprising trillions of microorganisms residing primarily in the gut, plays a crucial role in maintaining immune homeostasis and defending against pathogens as shown in Figure 3. Here’s an assessment of its role in preventing and treating infectious diseases caused by viruses, bacteria, and fungi.

### 2.32. Viral infections

#### 2.32.1. Prevention

Modulating the gut microbiota can enhance antiviral immunity by promoting the production of antiviral peptides and modulating immune responses. Probiotics and prebiotics have shown promise in reducing the risk of viral infections, including respiratory viruses like influenza and coronaviruses.^[22]^

#### 2.32.2. Treatment

While microbiota modulation alone may not directly treat viral infections, it can support the immune system’s response to viral pathogens. FMT has been explored as a potential adjunct therapy for certain viral infections, although research in this area is still in its infancy.

### 2.33. Bacterial infections

#### 2.33.1. Prevention

A balanced gut microbiota helps prevent colonization and overgrowth of pathogenic bacteria by competing for resources and producing antimicrobial substances. Probiotics, particularly strains of Lactobacillus and Bifidobacterium, have been shown to reduce the risk of bacterial infections such as Clostridioides difficile (C. difficile) and urinary tract infections.^[23]^

#### 2.33.2. Treatment

FMT has emerged as a highly effective treatment for recurrent C. difficile infection, with cure rates exceeding 90%. Additionally, probiotics have been investigated as adjunct therapies for various bacterial infections, although their efficacy can vary depending on the specific strain and infection.

### 2.34. Fungal infections

#### 2.34.1. Prevention

The gut microbiota plays a crucial role in preventing fungal overgrowth by maintaining a balanced microbial community and producing antifungal compounds. Probiotics and prebiotics have been explored for their potential to prevent fungal infections, particularly in immunocompromised individuals at risk of invasive candidiasis.

#### 2.34.2. Treatment

While less studied compared to bacterial and viral infections, microbiota modulation may have the potential to prevent and treat fungal infections. FMT has shown promise in restoring gut microbiota diversity and reducing the risk of fungal infections in certain patient populations.

Overall, microbiota modulation holds promise as a complementary approach to preventing and treating infectious diseases across various pathogens. However, more research is needed to elucidate the specific mechanisms involved, identify optimal interventions, and determine their efficacy in different clinical settings. Additionally, personalized approaches considering individual microbiota profiles and host factors are likely to enhance the effectiveness of microbiota-based interventions in infectious disease management.^[22]^

### 2.35. Effects: addressing safety considerations, adverse effects, and long-term implications of microbiota-modulating agents on human health

Microbiota-modulating agents, including probiotics, prebiotics, synbiotics, and fecal microbiota transplantation (FMT), have gained significant attention for their potential to positively impact human health by manipulating the composition and function of the gut microbiota. However, like any medical intervention, these agents come with safety considerations, potential adverse effects, and long-term implications that must be carefully evaluated.^[23]^

### 2.36. Safety considerations

Probiotics: Generally regarded as safe (GRAS) by regulatory agencies when consumed in recommended doses. However, in immunocompromised individuals or those with underlying health conditions, there’s a risk of infection or sepsis, particularly with strains that are opportunistic pathogens.

Prebiotics: Safe for most individuals when consumed within recommended doses. However, excessive intake can lead to gastrointestinal discomfort, bloating, and flatulence.

Synbiotics: Safety profile similar to probiotics and prebiotics individually, but interactions between components should be considered.^[10]^

FMT: Considered safe for certain indications, such as recurrent Clostridioides difficile infection, but there’s a risk of transmitting infectious agents or inducing adverse immune responses.

### 2.37. Adverse effects

Probiotics: Rare adverse effects include gastrointestinal symptoms (e.g., bloating, diarrhea), systemic infections (especially in immunocompromised individuals), and antibiotic resistance transfer.

Prebiotics: Generally well-tolerated, but excessive intake can lead to gastrointestinal symptoms.

Synbiotics: Adverse effects primarily relate to those of probiotics and prebiotics individually.

FMT: Potential adverse effects include gastrointestinal symptoms, infections, and immune reactions.

### 2.38. Long-term implications

Probiotics: Long-term effects are still being studied. Concerns include alterations in gut microbiota composition and function, potential for antibiotic resistance transfer, and effects on immune function.

Prebiotics: Long-term effects may include changes in gut microbiota composition and metabolic activity, which could have implications for overall health.

Synbiotics: Long-term implications are similar to those of probiotics and prebiotics individually, but the combined effects warrant further investigation.

FMT: Long-term effects are not well understood, including potential alterations in host microbiota, immune function, and metabolic health.

Microbiota-modulating agents hold promise for improving human health, their safety, adverse effects, and long-term implications should be carefully considered and studied further, especially in vulnerable populations and when used over extended periods. Close monitoring and appropriate regulation are essential to ensure their safe and effective use in clinical practice.

### 2.39. Future directions and innovative therapeutic strategies

Highlighting promising avenues for future research and development of novel microbiota-modulating agents, including microbial-based therapies, phage therapy, and microbiome engineering. Exploring future directions and innovative therapeutic strategies in the realm of microbiota modulation is an exciting area of research with significant potential for advancing healthcare. Let’s delve into some promising avenues for future development:

### 2.40. Microbial-based therapies

#### 2.40.1. Fecal Microbiota Transplantation (FMT)

FMT involves transferring fecal material from a healthy donor into the gastrointestinal tract of a recipient to restore a healthy microbial community. Future research may focus on standardizing protocols, identifying optimal donor characteristics, and exploring its efficacy in various disease states beyond Clostridium difficile infection, such as inflammatory bowel disease (IBD), metabolic disorders, and neurodegenerative diseases.

#### 2.40.2. Synthetic Microbial Consortia

Designing synthetic microbial consortia with defined compositions and functions holds promise for tailored therapeutic interventions. Future efforts may involve leveraging advances in synthetic biology, computational modeling, and high-throughput screening to engineer microbial communities that exert specific therapeutic effects, such as enhanced production of beneficial metabolites or targeted pathogen suppression.^[21]^

### 2.41. Phage therapy

#### 2.41.1. Bacteriophage Engineering

Advances in phage isolation, characterization, and genetic engineering offer opportunities to develop customized phage cocktails targeting specific pathogenic bacteria while preserving the native microbiota. Future research may focus on elucidating phage-host interactions, optimizing phage delivery methods, and overcoming challenges related to bacterial resistance and phage pharmacokinetics.

#### 2.41.2. Phage-Mediated Modulation of Microbiota

Beyond pathogen eradication, phages can modulate the composition and function of the microbiota. Future studies may explore the use of phages to selectively target and manipulate key microbial species involved in dysbiosis-associated diseases, such as IBD, obesity, and allergic disorders.^[22]^

### 2.42. Microbiome engineering

#### 2.42.1. Precision Microbiome Editing

CRISPR-based technologies enable precise manipulation of microbial genomes, offering potential avenues for engineering therapeutic microbiota with enhanced functionalities or altered community structures. Future research may focus on developing safe and efficient methods for microbiome editing, addressing concerns related to off-target effects and unintended ecological consequences.^[23]^

#### 2.42.2. Host-Microbiome Interactions

Deepening our understanding of host-microbiome interactions is crucial for designing targeted interventions. Future studies may employ systems biology approaches, including multi-omics profiling and computational modeling, to unravel complex host-microbiome dynamics and identify novel therapeutic targets.^[23]^

### 2.43. Microbiota-targeted drug development

#### 2.43.1. Microbiome-Modulating Drugs

Novel therapeutics designed to modulate the microbiota composition or activity hold promise for treating various diseases. Future drug development efforts may involve screening natural compounds, small molecules, and biologics for their microbiota-modulating effects and optimizing their pharmacokinetic properties for clinical use.^[22]^

### 2.44. Microbiota-directed prodrugs

Prodrugs that require microbial metabolism for activation represent an innovative approach for site-specific drug delivery and enhanced therapeutic efficacy. Future research may explore the design and optimization of microbiota-directed prodrugs for targeted treatment of gastrointestinal disorders, infections, and systemic diseases. Future research directions in microbiota modulation encompass a diverse array of approaches, ranging from microbial-based therapies and phage therapy to microbiome engineering and microbiota-targeted drug development. Continued innovation in these areas holds the potential to revolutionize healthcare by harnessing the therapeutic potential of the human microbiota.^[23]^

### 2.45. Regulatory considerations and market trends

Analyzing regulatory challenges, market dynamics, and healthcare policies influencing the development, approval, and commercialization of microbiota-modulating agents requires a comprehensive understanding of the evolving landscape in both the regulatory and market spheres. One of the primary challenges lies in defining and classifying microbiota-modulating agents. Depending on their mode of action, they might be classified as drugs, biologics, medical foods, or supplements, each of which has its own regulatory pathway. Demonstrating the safety and efficacy of these agents poses challenges due to the complex interactions within the gut microbiota and their systemic effects. Regulatory bodies often require extensive preclinical and clinical data to assess these aspects. Ensuring the quality and consistency of microbiota-modulating agents, particularly probiotics and fecal microbiota transplant (FMT) products, is a regulatory challenge. Standardization of manufacturing processes and quality control measures is crucial. There’s a growing awareness of the role of the gut microbiota in health and disease, driving demand for microbiota-modulating agents. Consumers are seeking alternatives to traditional pharmaceuticals, leading to a rise in the use of probiotics, prebiotics, and FMT. Advancements in microbiome research are driving innovation in the development of new microbiota-modulating agents. This includes personalized approaches targeting specific microbiota profiles associated with various diseases. The market is becoming increasingly competitive with the entry of new players, including pharmaceutical companies, biotechs, and startups, all vying for a share of the microbiota-modulating market.^[23]^

### 2.46. Healthcare policies

Reimbursement policies for microbiota-modulating agents vary across regions. Establishing reimbursement mechanisms for novel therapies like FMT remains a challenge in many healthcare systems. There’s a push for regulatory harmonization to streamline the approval process for microbiota-modulating agents across different regions. Efforts are underway to establish clear guidelines and standards for their development and commercialization. There’s increasing interest in developing microbiome-based diagnostics to guide the use of microbiota-modulating agents. This includes biomarkers for patient stratification and monitoring treatment response. Advances in synthetic biology are enabling the development of engineered microbiota with specific therapeutic functions, opening up new possibilities for targeted interventions. Navigating the regulatory landscape and market dynamics for microbiota-modulating agents requires addressing complex challenges related to safety, efficacy, standardization, and reimbursement, while also leveraging emerging trends in microbiome research and innovation. Close collaboration between industry, regulatory agencies, healthcare providers, and researchers is essential to drive the development and commercialization of effective microbiota-modulating therapies. Analyzing regulatory challenges, market dynamics, and healthcare policies influencing the development, approval, and commercialization of microbiota-modulating agents requires a comprehensive understanding of the evolving landscape in both the regulatory and market spheres.^[10]^

### 2.47. Regulatory challenges

Demonstrating the safety and efficacy of these agents poses challenges due to the complex interactions within the gut microbiota and their systemic effects. Regulatory bodies often require extensive preclinical and clinical data to assess these aspects. Ensuring the quality and consistency of microbiota-modulating agents, particularly probiotics and fecal microbiota transplant (FMT) products, is a regulatory challenge. Standardization of manufacturing processes and quality control measures is crucial. There’s a growing awareness of the role of the gut microbiota in health and disease, driving demand for microbiota-modulating agents. Consumers are seeking alternatives to traditional pharmaceuticals, leading to a rise in the use of probiotics, prebiotics, and FMT. Advancements in microbiome research are driving innovation in the development of new microbiota-modulating agents. This includes personalized approaches targeting specific microbiota profiles associated with various diseases. The market is becoming increasingly competitive with the entry of new players, including pharmaceutical companies, biotechs, and startups, all vying for a share of the microbiota-modulating market.^[24]^

### 2.48. Healthcare policies

Reimbursement policies for microbiota-modulating agents vary across regions. Establishing reimbursement mechanisms for novel therapies like FMT remains a challenge in many healthcare systems. There’s a push for regulatory harmonization to streamline the approval process for microbiota-modulating agents across different regions. Efforts are underway to establish clear guidelines and standards for their development and commercialization. There’s increasing interest in developing microbiome-based diagnostics to guide the use of microbiota-modulating agents. This includes biomarkers for patient stratification and monitoring treatment response. Advances in synthetic biology are enabling the development of engineered microbiota with specific therapeutic functions, opening up new possibilities for targeted interventions. Navigating the regulatory landscape and market dynamics for microbiota-modulating agents requires addressing complex challenges related to safety, efficacy, standardization, and reimbursement, while also leveraging emerging trends in microbiome research and innovation. Close collaboration between industry, regulatory agencies, healthcare providers, and researchers is essential to drive the development and commercialization of effective microbiota-modulating therapies.^[25]^

### 2.49. The role of green biomaterials principles in microbiota modulation

Green biomaterials principles in microbiota modulation is one of the upcoming ideas that fits into the system of caring the health of microbes by controlling the variety and function of the microbial communities.^[26]^ Microbiota modulation, based on changing the microbial communities within the human body, provides several facilities for health, agriculture, and environmental sustainability.^[27]^ Regulation of the microbiota has recently proved to have the potential to revolutionize gastrointestinal health diagnostics and therapy.^[28]^ Thus a path for personalized medicine where treatments can be modified in accordance with specific microbiome composition is paved. In addition, the microbiota-manipulating agents have the potential to face overactive immune systems and autoimmune diseases, thus, laying out another route for therapeutic interventions.^[29]^ The range of microbiota manipulation is not limited to gut health but extends to affect neurological conditions, metabolic syndrome, infectious diseases and regulation. For example, studies indicate a relationship between the gut microbiome and neurological disorders including depression, anxiety, Parkinson disease, and autism spectrum disorders. By steering the gut microbiome through different ways, eating habits improvements, probiotics and fecal microbiota transplantation, could be promising methods for managing these disorders.^[30]^ The 3 key principles of green biomaterials are sustainability, environmental friendliness and other specific principles. Implementation of these principles in microbiota modulation research and therapies gives a room for the development of the green technologies aimed at the reduction of the negative environmental repercussions.^[12]^ In spite of the fact of the possible facilitation, translating microbiota modulation science into clinical practice is a challenge. Examples include working on standardized protocols, improved therapy systems and continuous safety monitoring. Moreover, safety issues and the occurrence of adverse reactions, which may be stronger among specific groups of people, should be given serious attention.^[31]^ Throughput monitoring, control as well as compliance to strict quality measures are key to ensuring the security and efficiency of microbiota-modulating treatments. The advancing area of microbiota modulation entails creative therapies, such as microbiome-based therapies, phage therapy, the microbiome engineering, and drugs for the microbiota targeting. These approaches have a potential of developing customized therapies that can counter disease progression and health complications, resulting in the highest treatment outcome.^[3]^ The regulatory bottlenecks as well as the market dynamics are strong factors in the production and commercialization of microbiota modification drugs. Collaborations among the industrial players, the regulator, the healthcare providers and the researchers should be an important focus to guide safety, effectiveness, quality and reimbursement policies. Creating a regulatory system that would encourage the development of microbiota modulation innovation is imperative for patients in reaching new therapies as well as establishing a sustainable microbiota modulation field.^[12]^ Therefore, the integration of green biomaterials as microbia modulation principles into research and therapy shows enormous potential to change the health care not only for many health disorders but also to create a green environment. Nevertheless the identification problems related to standardization, safety, regulation, and cooperation are the main obstacles to speeding up microbiota modulation for improving human health and well-being.

### 2.50. The role of different biomaterials and materials in microbiota modulation

Recent innovations in biomaterials and materials bring about cutting-edge area in health care and environmental conservation. This field requires a diverse range of methods including dietary changes and complex genome editing methods. The benefits are versatile and can be felt by human health, agricultural practices, and environmental sustainability. In the field of gastrointestinal diseases major progress and achievements have been made in terms of diagnostic accuracy and specific therapy development, leading to personalized medicine.^[32]^ Microbial-modulating agents hold the key to the novel approach of correcting the imbalanced immune system by restoring the autoimmune disorders. Nonetheless, the translation of research to the clinical setting is not without some hurdles that would need consolidated approaches to protocol standardization, therapy optimization, and monitoring for integrated safety.^[31]^ Moreover, the microbiota manipulation also extends into areas like the neurological disorders, metabolic syndrome, and infectious diseases and might have regulatory consequences.^[3]^ The intricate connection between gut microbiota and neurodevelopment in these disorders suggest their involvement in neurological disorders such as depression, anxiety, Parkinson disease, and autism spectrum disorders.^[32]^ The data shows that the gut microbiome can be affected positively by approaches such as diet and lifestyle changes, the use of probiotics, and the procedure of fecal microbiota transplantation which in turn can minimize the likelihood of mood swings, behavioral problems, and cognitive decline. The gut-brain axis which occurs as a target for psychotherapeutic solutions seeks to address neurological disorders and enhance the quality of life of the affected individuals.^[23]^ Gut microbiota alterations are strongly connected with the rise of metabolic syndrome, insulin resistance, obesity, cardiovascular disease, and Type 2 diabetes. The gut microbiota can be targeted through dieting and microbial treatments that represent a hope for effective interventions against these multifaceted diseases and their comorbidities. There is a chance to implement microbiota manipulations in both preventing and treating infectious diseases caused by the diverse pathogens.^[31]^ Techniques covering probiotics, prebiotics, and fecal microbiota transplants reinforce the host’s immune response, increase the barrier of the mucosa, and discourage the colonization of pathogens and to weaken the infectious organisms. Nevertheless, safety concerns and adverse impacts on respect to the most vulnerable groups need a detailed examination. Comprehensive monitoring and control including safe and effective use of microbiota-modulating agents is mandatory in clinical settings. Considering the future, microbiome-based therapies, phage therapy, microbiome engineering, and drugs targeted at microbiota might be the next big direction for research.^[3]^ This patent approach of novel solutions have demonstrated high specificity to disease pathogenesis therefore improving the treatment outcomes through personalized interventions. Overcoming regulatory hurdles and market place challenges is very important for the microbiota-altering drugs to become the product offered on the market. Implementing a regulatory environment that fosters microbiota modulation innovation will help patients access advanced treatment options and ensure a steady economic endurance in this field.

Microbiota manipulation represents a significant opportunity for changing global health practices. It is critical to maximizing its potential.^[32]^ The problems related to standardization, optimization, security and supervision should be solved. Through analyzing the research quickly, coordinating integration, and application of innovative strategies, the microbiota modulation can optimize healthcare and promote worldwide health.

## 3. Conclusion

Microbiota modulation is a revolutionary practice that has far-reaching effects on human health and disease management. The strategies described in this review hold promise for modulating immune responses, managing gastrointestinal disorders, and treating a broad range of immunologically mediated diseases. Although there are continuing challenges, such as standardization and clinical translation, current research efforts will provide insights into the complex mechanisms and improve the treatment efficacy of microbiota-modulating agents in the future. This review emphasizes the importance of microbiota modulation in changing therapeutic paradigms and promoting personalized medicine.

## Author contributions

**Conceptualization:** Okechukwu Paul-Chima Ugwu, Esther Ugo Alum.

**Data curation:** Esther Ugo Alum, Michael Ben Okon.

**Formal analysis:** Okechukwu Paul-Chima Ugwu.

**Investigation:** Esther Ugo Alum.

**Methodology:** Okechukwu Paul-Chima Ugwu.

**Supervision:** Esther Ugo Alum, Emmanuel I. Obeagu.

**Validation:** Michael Ben Okon, Emmanuel I. Obeagu.

**Visualization:** Okechukwu Paul-Chima Ugwu, Esther Ugo Alum, Michael Ben Okon, Emmanuel I. Obeagu.

**Writing – original draft:** Okechukwu Paul-Chima Ugwu, Michael Ben Okon, Emmanuel I. Obeagu.

**Writing – review & editing:** Okechukwu Paul-Chima Ugwu, Esther Ugo Alum, Michael Ben Okon, Emmanuel I. Obeagu.
